# Prevalence and Factors Associated With Achlorhydria Among Patients With Gastric Cancer: A Two-Year Cross-Sectional Study

**DOI:** 10.7759/cureus.112730

**Published:** 2026-07-15

**Authors:** A. K. M. Mustakim Billah, Sajib Chandra Mandal, Rumana Parveen, Md. Rubaiyat Ibnea Hasan, Sumaiya Bente Jalil

**Affiliations:** 1 Department of Surgery, Dhaka Medical College and Hospital, Dhaka, BGD; 2 Department of Surgical Oncology, Faridpur Medical College and Hospital, Faridpur, BGD; 3 Department of Surgery, Gazaria Upazila Health Complex, Munshigonj, BGD; 4 Department of Child and Adolescent Psychiatry, National Institute of Mental Health, Dhaka, BGD

**Keywords:** achlorhydria, gastric acid secretion, gastric cancer, helicobacter pylori, intragastric ph

## Abstract

Background: Achlorhydria, characterized by absent or markedly impaired gastric acid secretion, has been associated with nutritional deficiencies, bacterial overgrowth, and chronic gastric mucosal injury. Altered gastric acid secretion may influence the clinical presentation and biological behavior of gastric cancer; however, evidence regarding its prevalence and associated factors among patients with gastric cancer in South Asia remains limited.

Objective: This study aimed to determine the prevalence of achlorhydria and evaluate its associations with demographic, laboratory, and clinicopathological characteristics among patients with histologically confirmed gastric cancer.

Methods: A hospital-based cross-sectional study was conducted at Dhaka Medical College Hospital, Bangladesh, from January 2022 to December 2023. One hundred consecutive adult patients with histologically confirmed gastric adenocarcinoma were enrolled. Achlorhydria was defined as a fasting intragastric pH >6.0 measured from gastric aspirate using a calibrated digital pH meter. Demographic, clinical, laboratory, and tumor-related data were collected. Categorical variables were compared using Pearson's chi-square or Fisher's exact test, as appropriate. Variables selected based on univariate analysis and clinical relevance were entered into a multivariable logistic regression model to identify factors independently associated with achlorhydria. Adjusted odds ratios (aORs) with 95% confidence intervals (CIs) were calculated.

Results: The mean age of participants was 60.8 ± 9.4 years, and 68.0% were male. Achlorhydria was identified in 38 patients, corresponding to a prevalence of 38.0% (95% CI: 28.5-48.3). Female sex (aOR: 5.10; 95% CI: 2.00-13.00; p = 0.001), hemoglobin ≤10 g/dL (aOR: 2.95; 95% CI: 1.17-7.48; p = 0.022), and proximal/body-predominant tumor location (aOR: 3.63; 95% CI: 1.41-9.34; p = 0.007) were independently associated with achlorhydria. Long-term proton pump inhibitor use was associated with achlorhydria in univariate analysis but was not independently associated after multivariable adjustment (aOR: 2.12; 95% CI: 0.76-5.91; p = 0.15).

Conclusions: Achlorhydria was common, affecting more than one-third of patients with gastric cancer. Female sex, anemia, and proximal/body-predominant tumor location were independently associated with achlorhydria. Given the cross-sectional design, these findings should be interpreted as associations rather than causal relationships. Larger prospective multicenter studies incorporating comprehensive assessment of gastric mucosal pathology and gastric acid secretory function are warranted to clarify the temporal relationship and clinical significance of achlorhydria in gastric cancer.

## Introduction

Gastric carcinoma remains a major public health concern because of its persistently high mortality burden worldwide. According to GLOBOCAN estimates, gastric cancer ranks among the leading causes of cancer-related death globally, particularly in Asian countries where incidence rates remain high [1]. The development of gastric cancer results from a multifaceted interplay of environmental, microbial, genetic, and host-related factors [2,3].

Alterations in gastric acid secretion have long been recognized as important contributors to gastric physiology and pathology. Normal gastric acid secretion is essential for efficient digestion, absorption of key nutrients, maintenance of microbial balance within the gastrointestinal tract, and protection against enteric pathogens [4]. Achlorhydria refers to absent or markedly reduced hydrochloric acid secretion by gastric parietal cells and may occur secondary to chronic atrophic gastritis, autoimmune gastritis, advanced gastric mucosal injury, or prolonged acid-suppressive therapy [5,6].

Previous investigations have linked impaired gastric acid secretion with iron deficiency, vitamin B12 depletion, bacterial overgrowth, and sustained elevations in serum gastrin levels [7,8]. In addition, longstanding achlorhydria has been observed in patients with pernicious anemia and chronic atrophic gastritis, both of which are recognized risk conditions for gastric neoplasia [9]. Nevertheless, the prevalence and clinical correlates of achlorhydria among patients already diagnosed with gastric cancer remain insufficiently characterized.

Proton pump inhibitors (PPIs) are widely prescribed and may induce profound acid suppression during prolonged use [10]. While several observational studies have suggested associations between long-term acid suppression and gastric mucosal changes, the relationship between gastric acid secretion and tumor characteristics remains incompletely understood [11].

The present study aimed to determine the prevalence of achlorhydria among patients with histologically confirmed gastric cancer and to evaluate its associations with demographic, laboratory, and clinicopathological features, including tumor characteristics and clinical factors, using a cross-sectional study design.

## Materials and methods

Study design and setting

This hospital-based cross-sectional study was conducted in the Department of Surgery, Dhaka Medical College Hospital, Bangladesh, over a two-year period from January 2022 to December 2023. The study was reported according to the Strengthening the Reporting of Observational Studies in Epidemiology (STROBE) Statement guidelines for reporting observational studies [12].

Participants

Consecutive adult patients with histologically confirmed gastric adenocarcinoma were screened for eligibility. A total of 108 patients were assessed for eligibility. Eight patients were excluded (five declined participation and three had incomplete gastric pH measurements), resulting in a final study population of 100 participants. 

Inclusion criteria

Adult patients (≥18 years) with histologically confirmed gastric adenocarcinoma who provided written informed consent were eligible for inclusion. Information regarding long-term PPI use was collected as a study variable but was not required for enrollment.

Exclusion criteria

Patients were excluded if they were younger than 18 years; had a previous gastrectomy; had severe sepsis, septic shock, or hemodynamic instability; were unable to undergo gastric pH assessment, including failure to obtain an adequate gastric aspirate for pH measurement; had incomplete clinical or laboratory data required for analysis; or refused or were unable to provide written informed consent.

Sample size calculation

The sample size was calculated using the single-population proportion formula:

$n = \frac{Z^2pq}{d^2}$

where Z = 1.96 for a 95% confidence interval (CI), p = 0.50, $q = 1 - p$ = 0.50, d = 0.10, $n = \frac{(1.96)^2 \times 0.50 \times 0.50}{(0.10)^2}$, and n = 96.04. The minimum required sample size was therefore 96 participants. To compensate for incomplete data and exclusions, 100 patients were recruited.

Definition of achlorhydria

After overnight fasting, gastric juice was aspirated through a nasogastric tube and immediately analyzed using a calibrated digital pH meter. Achlorhydria was defined as fasting intragastric pH >6.0 [5,13].

Study outcomes

Primary Outcome

The primary outcome was the prevalence of achlorhydria among patients with histologically confirmed gastric adenocarcinoma. 

Secondary Outcomes

The secondary outcomes were the identification of demographic, clinical, laboratory, and tumor-related factors independently associated with achlorhydria. Variables evaluated included age, sex, smoking history, long-term PPI use, hemoglobin level, *Helicobacter pylori* infection status, family history of gastric cancer, and tumor location. Associations between these variables and achlorhydria were examined using univariate analyses followed by multivariable logistic regression to identify factors independently associated with achlorhydria.

Data collection

Data were collected using a structured questionnaire that included information on age, sex, occupation, socioeconomic status, smoking history, family history of gastric cancer, *Helicobacter pylori* infection, peptic ulcer disease, gastroesophageal reflux disease, laboratory findings, upper gastrointestinal endoscopy findings, and histopathology reports.

Measurement of intragastric pH

Patients underwent overnight fasting for at least eight hours before intragastric pH assessment. A nasogastric tube was inserted using standard clinical technique, and gastric juice was aspirated after confirmation of intragastric placement based on the aspiration of gastric contents. The aspirated sample was analyzed immediately with a calibrated handheld digital pH meter, following the manufacturer's instructions, to minimize pH changes from prolonged exposure to ambient conditions. The pH meter was calibrated before each measurement using standard buffer solutions (pH 4.0 and 7.0). All patients discontinued PPIs for at least seven days and H2-receptor antagonists for at least 48 hours before undergoing ambulatory 24-hour esophageal pH monitoring. All enrolled participants complied with this medication washout protocol, and no patient underwent pH monitoring while receiving acid-suppressive therapy. Information on long-term PPI use was recorded as a study variable and included in the statistical analysis due to its potential influence on gastric acidity.

Assessment of *H. pylori*

*H. pylori* infection was assessed before initiation of any treatment using the rapid urease test (RUT). During upper gastrointestinal endoscopy, biopsy specimens were obtained from both the gastric antrum and corpus for all participants. The same standardized diagnostic protocol was applied uniformly throughout the study.

Statistical analysis

Continuous variables were summarized as mean ± standard deviation, whereas categorical variables were presented as frequencies and percentages. Exact binomial 95% CIs were calculated for prevalence estimates. For categorical variables, Pearson's chi-square (χ²) statistic or Fisher's exact test was reported as appropriate. For continuous variables, independent-samples t-tests were used when comparing means. Corresponding test statistics (χ² or t values) together with p values were reported. Variables with a p value of <0.20 in the univariate analysis were considered for inclusion in the multivariable logistic regression model. In addition, long-term PPI use was included a priori irrespective of its statistical significance because of its well-established biological effect on gastric acid secretion and its potential to confound the association between clinicopathological factors and achlorhydria. Adjusted odds ratios (aORs) with 95% CIs were calculated. Model calibration was assessed using the Hosmer-Lemeshow goodness-of-fit test, while explanatory performance was evaluated using the Nagelkerke R² statistic. For regression analysis, tumor location was dichotomized as proximal/body-predominant (body, cardia/fundus, or body plus antrum involvement) vs. distal-predominant (antrum/pylorus). Statistical analyses were performed using IBM Statistical Package for the Social Sciences Statistics for Windows, version 26.0 (IBM Corp., Armonk, NY). A two-sided p value of <0.05 was considered statistically significant.

Ethical considerations

Ethical approval was obtained from the Institutional Review Board of Dhaka Medical College Hospital (approval no. ERC-DMC/ECC/113/2021). Written informed consent was obtained from all participants before enrollment.

## Results

During the two-year study period, 108 patients with histologically confirmed gastric cancer were screened for eligibility. Eight patients were excluded, including five who declined participation and three who had incomplete gastric pH measurements. Consequently, 100 patients were included in the final analysis. No missing data were identified for the variables included in the study.

The mean age of the participants was 60.8 ± 9.4 years (range: 38-82 years). The largest proportion of patients belonged to the 60-69-year age group (36.0%), followed by the 50-59-year age group (26.0%), ≥70 years (20.0%), and <50 years (18.0%). Male participants constituted 68.0% (n = 68) of the study population, while female participants accounted for 32.0% (n = 32), resulting in a male-to-female ratio of approximately 2.1:1.

Smoking history was present in 58.0% (n = 58) of participants. Long-term PPI use exceeding 12 months was documented in 71.0% (n = 71) of patients. Anemia was common, with 56.0% (n = 56) of participants demonstrating hemoglobin levels ≤10 g/dL. *H. pylori* infection was detected in 14.0% (n = 14) of patients, while a family history of gastric cancer was reported by 8.0% (n = 8) of the study population (Table 1).

**Table 1:** Baseline demographic and clinical characteristics of the study population (n = 100) Values are presented as frequencies (percentages) unless otherwise indicated. Age is expressed as mean ± SD. Long-term PPI use was defined as continuous use for more than 12 months. Smoking history included both current and former smokers. Hemoglobin concentration was categorized using a threshold of ≤10 g/dL. Helicobacter pylori positivity was determined by histopathological examination and/or rapid urease testing performed during upper gastrointestinal endoscopy. Family history of gastric cancer refers to a first-degree relative with a documented diagnosis of gastric malignancy. The total study population is 100 participants SD: standard deviation; PPI: proton pump inhibitor

Variable	Frequency (%)
Age (years)	
<50	18 (18.0)
50-59	26 (26.0)
60-69	36 (36.0)
≥70	20 (20.0)
Mean ± SD	60.8 ± 9.4
Sex	
Male	68 (68.0)
Female	32 (32.0)
Smoking history	58 (58.0)
Long-term PPI use (>12 months)	71 (71.0)
Hemoglobin ≤10 g/dL	56 (56.0)
*Helicobacter pylori* positive	14 (14.0)
Family history of gastric cancer	8 (8.0)

Upper gastrointestinal endoscopy revealed that distal gastric tumors involving the antrum or pylorus were the most frequent lesions, accounting for 52.0% (n = 52) of cases. Tumors involving the gastric body alone were identified in 24.0% (n = 24), whereas cardia or fundus lesions were observed in 14.0% (n = 14). Combined involvement of the body and antrum was present in 10.0% (n = 10) of patients.

Histopathological examination demonstrated that intestinal-type adenocarcinoma was the predominant subtype, accounting for 61.0% (n = 61) of cases, while diffuse-type adenocarcinoma was identified in 39.0% (n = 39). Regarding gross morphology, ulceroproliferative lesions represented the most common endoscopic appearance (44.0%, n = 44), followed by fungating lesions (36.0%, n = 36) and ulcerative lesions (20.0%, n = 20) (Table 2).

**Table 2:** Endoscopic and histopathological characteristics Values are presented as frequency (percentage). Tumor location was determined based on upper gastrointestinal endoscopy and categorized according to the primary anatomical site of involvement. Distal tumors included lesions located in the gastric antrum and pylorus. Histopathological classification into intestinal-type and diffuse-type adenocarcinoma was performed according to the Lauren V. Ackerman/Lauren classification system. Gross morphological appearance was assessed endoscopically and categorized as ulceroproliferative, fungating, or ulcerative lesions. Percentages are based on the total study population (n = 100)

Variable	Frequency (%)
Distal tumor (antrum/pylorus)	52 (52.0)
Body	24 (24.0)
Cardia/fundus	14 (14.0)
Body + antrum	10 (10.0)
Intestinal-type adenocarcinoma	61 (61.0)
Diffuse-type adenocarcinoma	39 (39.0)
Ulceroproliferative lesion	44 (44.0)
Fungating lesion	36 (36.0)
Ulcerative lesion	20 (20.0)

Based on fasting intragastric pH measurements, achlorhydria (defined as intragastric pH >6.0) was identified in 38 patients, yielding an overall prevalence of 38.0% (95% CI: 28.5-48.3). The remaining 62 patients (62.0%) had intragastric pH values ≤6.0 and were classified as nonachlorhydric.

Female patients demonstrated a significantly higher prevalence of achlorhydria compared with male patients. Among the 32 female participants, 22 (68.8%) had achlorhydria, whereas only 16 of 68 male patients (23.5%) were achlorhydric (χ² = 16.9, p<0.001). Anemia was also significantly associated with achlorhydria. Among patients with hemoglobin levels ≤10 g/dL, 28 of 56 (50.0%) exhibited achlorhydria compared with only 10 of 44 (22.7%) patients with hemoglobin levels >10 g/dL (χ² = 8.29, p = 0.004). Tumor location demonstrated a significant association with gastric acid status. Patients with proximal or body-predominant tumors showed a markedly higher prevalence of achlorhydria than those with distal tumors (54.2% vs. 23.1%, χ² = 9.81, p = 0.002). No significant associations were observed for smoking status (χ² = 1.57, p = 0.21), *H. pylori* infection (Fisher's exact p = 0.53), or family history of gastric cancer (Fisher's exact p > 0.99). Long-term PPI use was documented in 71% of study participants. Among patients with achlorhydria, 32 of 38 (84.2%) reported continuous PPI use for more than 12 months compared with 39 of 62 (62.9%) patients without achlorhydria. This association was statistically significant on univariate analysis (χ² = 5.08, p = 0.024). Because chronic acid-suppressive therapy may directly influence intragastric pH measurements, long-term PPI use was included in an exploratory multivariable analysis (Table 3).

**Table 3:** Association between clinicopathological variables and achlorhydria Data are presented as n (%). Pearson's chi-square test was used for categorical variables unless expected cell counts were <5, in which case Fisher's exact test was applied. Test statistics (χ²) and corresponding p values are shown. Achlorhydria was defined as fasting intragastric pH > 6.0, measured from gastric aspirate Hb: hemoglobin; PPI: proton pump inhibitor

Variable	Achlorhydria (n = 38)	No achlorhydria (n = 62)	Test	Statistic (χ²)	p value
Female sex	22 (57.9%)	10 (16.1%)	Chi-square	16.9	<0.001
Smoking	25 (65.8%)	33 (53.2%)	Chi-square	1.57	0.21
Long-term PPI use	32 (84.2%)	39 (62.9%)	Chi-square	5.08	0.024
Hb ≤10 g/dL	28 (73.7%)	28 (45.2%)	Chi-square	8.29	0.004
*Helicobacter pylori* positive	4 (10.5%)	10 (16.1%)	Fisher's exact	-	0.53
Proximal/body tumor	26 (68.4%)	22 (35.5%)	Chi-square	9.81	0.002
Family history	3 (7.9%)	5 (8.1%)	Fisher's exact	-	>0.99

Variables with a p value of <0.20 in the univariate analysis were entered into the multivariable logistic regression model. Long-term PPI use was additionally retained in the model because of its established clinical relevance as a determinant of gastric acid secretion. Consequently, the final model included age, sex, smoking status, long-term PPI use, hemoglobin level, *H. pylori *infection status, and tumor location. After adjustment for potential confounders, female sex remained the strongest independent predictor of achlorhydria (aOR: 5.10; 95% CI: 2.00-13.00; p = 0.001). Female patients had approximately fivefold higher odds of having achlorhydria than male patients. Anemia (hemoglobin ≤10 g/dL) was also independently associated with achlorhydria, with affected patients demonstrating nearly threefold increased odds compared with nonanemic patients (aOR: 2.95; 95% CI: 1.17-7.48; p = 0.022). Similarly, proximal or body-predominant tumor location remained a significant independent predictor of achlorhydria (aOR: 3.63; 95% CI: 1.41-9.34; p = 0.007). In contrast, smoking history (aOR: 1.27; 95% CI: 0.56-2.89; p = 0.57), *H. pylori* infection (aOR: 0.75; 95% CI: 0.18-3.20; p = 0.70), and age ≥60 years (aOR: 1.42; 95% CI: 0.61-3.32; p = 0.42) did not demonstrate statistically significant independent associations with achlorhydria. Long-term PPI use showed a trend toward increased odds of achlorhydria; however, the association was not statistically significant after adjustment for confounding variables (adjusted OR: 2.12; 95% CI: 0.76-5.91; p = 0.15). Therefore, prolonged PPI exposure was not considered an independent predictor of achlorhydria in this cohort. The final regression model demonstrated acceptable calibration, with a Hosmer-Lemeshow goodness-of-fit p value of 0.71. The model explained approximately 29% of the variance in achlorhydria status (Nagelkerke R² = 0.29) and correctly classified 74% of study participants (Table 4).

**Table 4:** Multivariable logistic regression analysis for predictors of achlorhydria Multivariable logistic regression was performed to identify independent predictors of achlorhydria among patients with gastric carcinoma. Variables included in the model were age, sex, smoking status, hemoglobin level, Helicobacter pylori infection status, and tumor location. β coefficients represent the estimated regression coefficients, and adjusted ORs were calculated by exponentiating the β coefficients. CIs are reported at the 95% level. Statistical significance was defined as p < 0.05. Female sex, hemoglobin ≤10 g/dL, and proximal/body tumor location were independently associated with increased odds of achlorhydria Hosmer-Lemeshow p = 0.71 Nagelkerke R² = 0.29 Classification accuracy = 74% Hb: hemoglobin; SE: standard error; OR: odds ratio; CI: confidence interval; PPI: proton pump inhibitor

Variable	β coefficient (SE)	Adjusted OR	95% CI	p value
Female sex	1.63 (0.48)	5.10	2.00-13.00	0.001
Hb ≤10 g/dL	1.08 (0.47)	2.95	1.17-7.48	0.022
Proximal/body tumor	1.29 (0.48)	3.63	1.41-9.34	0.007
Long-term PPI use	0.75 (0.52)	2.12	0.76-5.91	0.15
Smoking	0.24 (0.42)	1.27	0.56-2.89	0.57
Helicobacter pylori positivity	-0.29 (0.71)	0.75	0.18-3.20	0.70
Age ≥60 years	0.35 (0.43)	1.42	0.61-3.32	0.42

Overall, achlorhydria was identified in more than one-third of patients with gastric cancer. Female sex, anemia, and proximal or body-predominant tumor location were independently associated with achlorhydria after multivariable adjustment. Conversely, smoking history, *H. pylori* infection, family history of gastric cancer, and older age were not independently associated with achlorhydria in this cohort. These findings indicate that alterations in gastric acid secretion are common among patients with gastric cancer and may be related to specific demographic and tumor-related characteristics.

## Discussion

In the current cohort, achlorhydria was identified in 38% of individuals diagnosed with gastric cancer. This prevalence is substantially higher than estimates reported in healthy populations, suggesting that impaired gastric acid secretion is common among patients with gastric malignancy [13]. Previous investigations among patients with chronic atrophic gastritis, pernicious anemia, and advanced gastric mucosal disease have similarly demonstrated substantially elevated rates of achlorhydria, supporting the biological relationship between progressive gastric mucosal damage and impaired acid production [14,15] . Our findings therefore reinforce the concept that disruption of normal gastric secretory function is common among patients with gastric malignancy.

Female sex emerged as the strongest independent predictor of achlorhydria. Similar findings have been reported in studies of autoimmune gastritis and pernicious anemia, conditions characterized by progressive destruction of acid-secreting parietal cells [16]. The observed association between female sex and achlorhydria may reflect underlying biological mechanisms, including the higher prevalence of autoimmune gastritis among women and the greater susceptibility of the gastric body and fundus to parietal cell loss. However, autoimmune gastritis and other relevant biomarkers were not assessed in this study; therefore, these mechanisms remain speculative.

The association between anemia and fasting intragastric pH >6.0 should also be interpreted cautiously. Although impaired gastric acid secretion may contribute to iron and vitamin B12 deficiency, anemia in patients with gastric cancer is frequently multifactorial and may also result from chronic gastrointestinal blood loss, cancer-related systemic inflammation, nutritional deficiencies, cachexia, or reduced dietary intake. The high prevalence of anemia observed in this study is consistent with previous reports among patients with gastric malignancy [17].

The independent association between proximal/body-predominant tumors and fasting intragastric pH >6.0 is biologically plausible because the gastric body and fundus contain the majority of acid-secreting parietal cells [18]. Nevertheless, fasting intragastric pH may also be influenced by tumor-related destruction of oxyntic mucosa, preexisting atrophic gastritis, chronic acid-suppressive therapy, or other unmeasured biological factors. Therefore, the present findings do not establish whether impaired gastric acid secretion preceded gastric cancer or developed as a consequence of the underlying disease.

No significant association was observed between *H. pylori *infection and achlorhydria. However, the prevalence of *H. pylori* infection in our cohort (14%) was lower than that reported in many gastric cancer studies [19,20]. Although all patients underwent *H. pylori* testing before treatment using a standardized RUT on biopsy specimens obtained from both the antrum and corpus, several factors may have contributed to the underestimation of the true prevalence. Previous long-term PPI use may have reduced bacterial density and the sensitivity of the RUT despite protocol-mandated medication washout before intragastric pH assessment. In addition, undocumented prior eradication therapy or antibiotic exposure, advanced gastric atrophy or intestinal metaplasia, and tumor-related mucosal changes may have resulted in false-negative findings. Therefore, the relatively low *H. pylori* positivity rate should be interpreted with caution.

Long-term PPI therapy has been associated with an increased risk of gastric cancer in several studies, particularly among patients with prior *H. pylori* infection [21,22]. Proposed mechanisms include profound acid suppression, hypergastrinemia, enterochromaffin-like cell hyperplasia, and progression of corpus-predominant atrophic gastritis. However, these associations remain controversial because most available evidence is observational and susceptible to confounding by indication, reverse causation, and residual bias. Consequently, a causal relationship between chronic PPI use and gastric carcinogenesis has not been established. In the present study, long-term PPI use was included in the multivariable model because of its established biological effect on gastric acid secretion. The relatively modest sample size and limited number of achlorhydria events may have reduced the precision of multivariable adjustment. Although it was not independently associated with elevated fasting intragastric pH after adjustment, this finding should be interpreted cautiously and should not be considered evidence that long-term PPI exposure lacks clinical relevance.

The present study possesses several strengths, including standardized pH measurement, explicit achlorhydria definition, STROBE-compliant methodology, and multivariable logistic regression analysis. Several limitations should be acknowledged. First, the cross-sectional design prevents causal inference. Second, this was a single-center study, which may limit generalizability to broader populations. Third, serum gastrin levels, vitamin B12 concentrations, iron studies, and autoimmune markers were not assessed. Finally, although multivariable adjustment was performed, the relatively modest number of achlorhydria events may have limited the precision of regression estimates, and the findings should therefore be interpreted cautiously.

Several limitations should be acknowledged. First, the cross-sectional design precludes determination of temporal or causal relationships between achlorhydria and the associated factors identified. Second, this was a single-center study with a relatively modest sample size, which may limit the generalizability of the findings. Third, important mechanistic variables, including gastric atrophy, intestinal metaplasia, autoimmune gastritis markers, serum gastrin, pepsinogen profile, vitamin B12 concentration, and iron studies, were not assessed. Fourth, although multivariable logistic regression was performed and the model demonstrated acceptable calibration (Hosmer-Lemeshow p = 0.71), the relatively small number of achlorhydria events (n = 38) in relation to the number of variables included in the model may have increased the risk of overfitting and reduced the precision and stability of the estimated odds ratios. Therefore, the observed associations should be interpreted with caution and require confirmation in larger prospective studies with sufficient event numbers and external validation.

The findings of this study should be interpreted in light of its cross-sectional design, which precludes determination of the temporal relationship between achlorhydria and gastric cancer. Consequently, the observed associations do not establish whether achlorhydria precedes gastric carcinogenesis or develops secondary to tumor-related mucosal changes. Our results suggest that achlorhydria may represent a marker of underlying gastric mucosal pathology, rather than a causal factor in gastric cancer development. This interpretation should be considered hypothesis-generating and requires validation in larger prospective, multicenter studies incorporating detailed assessment of gastric atrophy, serum gastrin and pepsinogen concentrations, iron status, vitamin B12 levels, and standardized medication washout protocols.

## Conclusions

Achlorhydria was a common finding in this cohort, affecting more than one-third of patients with histologically confirmed gastric cancer. Female sex, anemia, and proximal/body-predominant tumor location emerged as significant independent predictors of achlorhydria, suggesting that impaired gastric acid secretion is associated with specific demographic and tumor-related characteristics. Although long-term PPI use was frequent among study participants and showed an association with achlorhydria on univariate analysis, it was not an independent predictor after multivariable adjustment. These findings highlight the complex interplay between gastric acid secretory dysfunction and gastric carcinogenesis and suggest that achlorhydria may represent a clinically relevant marker of underlying gastric mucosal pathology. Further large-scale, multicenter prospective studies incorporating gastric atrophy assessment, serum gastrin measurements, pepsinogen profiling, and detailed histopathological evaluation are warranted to clarify the pathophysiological mechanisms and prognostic implications of achlorhydria in patients with gastric cancer.

## References

[1] Bray F, Ferlay J, Soerjomataram I, Siegel RL, Torre LA, Jemal A (2018). Global cancer statistics 2018: GLOBOCAN estimates of incidence and mortality worldwide for 36 cancers in 185 countries. CA Cancer J Clin.

[2] Rawla P, Barsouk A (2019). Epidemiology of gastric cancer: global trends, risk factors and prevention. Prz Gastroenterol.

[3] Figueiredo C, Camargo MC, Leite M, Fuentes-Pananá EM, Rabkin CS, Machado JC (2017). Pathogenesis of gastric cancer: genetics and molecular classification. Molecular Pathogenesis and Signal Transduction by Helicobacter pylori. Current Topics in Microbiology and Immunology.

[4] Vannella L, Lahner E, Osborn J, Annibale B (2013). Systematic review: gastric cancer incidence in pernicious anaemia. Aliment Pharmacol Ther.

[5] Annibale B, Lahner E, Fave GD (2011). Diagnosis and management of pernicious anemia. Curr Gastroenterol Rep.

[6] Gomez Cifuentes JD, Sparkman J, Graham DY (2022). Management of upper gastrointestinal symptoms in patients with autoimmune gastritis. Curr Opin Gastroenterol.

[7] Ito T, Jensen RT (2010). Association of long-term proton pump inhibitor therapy with bone fractures and effects on absorption of calcium, vitamin B12, iron, and magnesium. Curr Gastroenterol Rep.

[8] Lahner E, Annibale B (2009). Pernicious anemia: new insights from a gastroenterological point of view. World J Gastroenterol.

[9] Strand DS, Kim D, Peura DA (2017). 25 years of proton pump inhibitors: a comprehensive review. Gut Liver.

[10] Cheung KS, Chan EW, Wong AY, Chen L, Wong IC, Leung WK (2018). Long-term proton pump inhibitors and risk of gastric cancer development after treatment for Helicobacter pylori: a population-based study. Gut.

[11] Brusselaers N, Wahlin K, Engstrand L, Lagergren J (2017). Maintenance therapy with proton pump inhibitors and risk of gastric cancer: a nationwide population-based cohort study in Sweden. BMJ Open.

[12] von Elm E, Altman DG, Egger M, Pocock SJ, Gøtzsche PC, Vandenbroucke JP (2007). The Strengthening the Reporting of Observational Studies in Epidemiology (STROBE) statement: guidelines for reporting observational studies. PLoS Med.

[13] Jannot AS, Girardeau Y, Chaussade S, Cerf-Bensussan N, Malamut G (2024). Increased risk of gastric cancer in relation with pernicious anaemia in patients with primary antibody deficiency: a nationwide case control study. Dig Liver Dis.

[14] Sipponen P, Maaroos HI (2015). Chronic gastritis. Scand J Gastroenterol.

[15] Thota V, Paravathaneni M, Bedi V, Branham M, Thirumaran R (2021). Rapid development of pernicious anemia unmasking underlying gastric adenocarcinoma. Cureus.

[16] Hershko C, Ronson A (2009). Iron deficiency, Helicobacter infection and gastritis. Acta Haematol.

[17] Kuipers EJ, Uyterlinde AM, Peña AS (1995). Long-term sequelae of Helicobacter pylori gastritis. Lancet.

[18] Jiang K, Jiang X, Wen Y, Liao L, Liu FB (2019). Relationship between long-term use of proton pump inhibitors and risk of gastric cancer: a systematic analysis. J Gastroenterol Hepatol.

[19] Mégraud F, Bessède E, Varon C (2015). Helicobacter pylori infection and gastric carcinoma. Clin Microbiol Infect.

[20] Talebi Bezmin Abadi A (2016). Helicobacter pylori and gastric cancer. Front Med (Lausanne).

[21] Abbas MK, Zaidi AR, Robert CA, Thiha S, Malik BH (2019). The safety of long-term daily usage of a proton pump inhibitor: a literature review. Cureus.

[22] Seo SI, Park CH, You SC (2021). Association between proton pump inhibitor use and gastric cancer: a population-based cohort study using two different types of nationwide databases in Korea. Gut.

